# Incidence of and predictors for antiseizure medication gaps in Medicare beneficiaries with epilepsy: a retrospective cohort study

**DOI:** 10.1186/s12883-022-02852-6

**Published:** 2022-09-01

**Authors:** Samuel W. Terman, Joshua D. Niznik, Geertruida Slinger, Willem M. Otte, Kees P. J. Braun, Carole E. Aubert, Wesley T. Kerr, Cynthia M. Boyd, James F. Burke

**Affiliations:** 1 Department of Neurology, University of Michigan, Ann Arbor, MI 48109 USA; 2 Division of Geriatric Medicine, Center for Aging and Health, School of Medicine, University of North Carolina At Chapel Hill, Chapel Hill, NC 27599 USA; 3 Division of Pharmaceutical Outcomes and Policy, Eshelman School of Pharmacy, University of North Carolina At Chapel Hill, Chapel Hill, NC 27599 USA; 4 Department of Child Neurology, University Medical Center Utrecht, Utrecht University, Utrecht, The Netherlands; 5 Department of General Internal Medicine, Inselspital, Bern University Hospital, University of Bern, Bern, Switzerland; 6 Institute of Primary Health Care (BIHAM), University of Bern, Mittelstrasse 43, 3012 Bern, Switzerland; 7 Division of Geriatric Medicine and Gerontology, Johns Hopkins University School of Medicine, Baltimore, MD 21224 USA; 8 Department of Neurology, the Ohio State University, Columbus, OH 43210 USA

**Keywords:** Epilepsy, Antiseizure medications, Administrative claims, Discontinuation

## Abstract

**Background:**

For the two-thirds of patients with epilepsy who achieve seizure remission on antiseizure medications (ASMs), patients and clinicians must weigh the pros and cons of long-term ASM treatment. However, little work has evaluated how often ASM discontinuation occurs in practice. We describe the incidence of and predictors for sustained ASM fill gaps to measure discontinuation in individuals potentially eligible for ASM withdrawal.

**Methods:**

This was a retrospective cohort of Medicare beneficiaries. We included patients with epilepsy by requiring International Classification of Diseases codes for epilepsy/convulsions plus at least one ASM prescription each year 2014–2016, and no acute visit for epilepsy 2014–2015 (i.e., potentially eligible for ASM discontinuation). The main outcome was the first day of a gap in ASM supply (30, 90, 180, or 360 days with no pills) in 2016–2018. We displayed cumulative incidence functions and identified predictors using Cox regressions.

**Results:**

Among 21,819 beneficiaries, 5191 (24%) had a 30-day gap, 1753 (8%) had a 90-day gap, 803 (4%) had a 180-day gap, and 381 (2%) had a 360-day gap. Predictors increasing the chance of a 180-day gap included number of unique medications in 2015 (hazard ratio [HR] 1.03 per medication, 95% confidence interval [CI] 1.01–1.05) and epileptologist prescribing physician (≥25% of that physician’s visits for epilepsy; HR 2.37, 95% CI 1.39–4.03). Predictors decreasing the chance of a 180-day gap included Medicaid dual eligibility (HR 0.75, 95% CI 0.60–0.95), number of unique ASMs in 2015 (e.g., 2 versus 1: HR 0.37, 95% CI 0.30–0.45), and greater baseline adherence (> 80% versus ≤80% of days in 2015 with ASM pill supply: HR 0.38, 95% CI 0.32–0.44).

**Conclusions:**

Sustained ASM gaps were rarer than current guidelines may suggest. Future work should further explore barriers and enablers of ASM discontinuation to understand the optimal discontinuation rate.

**Supplementary Information:**

The online version contains supplementary material available at 10.1186/s12883-022-02852-6.

## Background

Identifying candidates for long-term antiseizure medication (ASM) treatment represents a key aspect of epilepsy care. Seizures may lead to injuries and acute care use, jeopardize driving privileges, and worsen quality of life [1–4]. Accordingly, ASMs reduce morbidity and mortality by reducing seizures, [2, 3] and undertreatment is common [5]. However, long-term ASM treatment also has downsides. Adverse effects range from 10 to 40% in unstructured screening or up to 60–90% [6] in structured screenings, and correlate with worsened quality of life [1, 7–10] explaining up to a quarter of variation in quality of life [11]. Treatment may also be costly, [12] many ASMs exert drug interactions [13] or require monitoring, and epilepsy is fraught with overdiagnosis which would reduce the long-term benefit for some [14, 15].

Fortunately, two-thirds of patients with epilepsy become seizure-free on ASMs. For these patients, given the above tradeoffs, guidelines have suggested considering ASM discontinuation after 2 years of seizure-freedom [16] after a detailed assessment of risk factors for seizure recurrence [17]. Up to 70% will remain seizure-free even off ASMs, [18] ASM discontinuation can improve key patient-centered outcomes such as mood [19] and cognition, [20] and this is a population at low risk for sudden unexpected death in epilepsy (27-times less common in patients without a convulsion in the last year compared with a baseline of ~ 1 per 1000 person-years [21]).

While many cohorts have estimated post-withdrawal seizure relapse risk, [18] only limited literature [22–24] has focused on the implementation of discontinuation decisions in practice [25]. For instance, single-center work found that only one-third of seizure-free patients reported their physician had even mentioned the possibility of ASM discontinuation suggesting that discontinuation attempts may be too rare [26]. While there is no single known ‘optimal’ rate for how often patients should consider a discontinuation attempt, such work raises the hypothesis that discontinuation attempts are occurring more seldom than what guidelines would endorse. Work aimed at better understanding the frequency and predictors of real-world discontinuation would inform the degree to which guidelines are being implemented to identify opportunities to optimize medication use.

In this study, we leveraged a large longitudinal national US database linked with physician information to primarily describe the cumulative incidence of sustained “gaps” in ASM fills (which we define as timespans without ASM prescription supply) in patients with prevalent treated epilepsy. Secondarily, we described predictors of gaps.

## Methods

### Study design and dataset

This was a retrospective cohort study of people with epilepsy in a 20% random sample of fee-for-service Medicare administrative claims using data from 2014 to 2015 (baseline) and 2016–2018 (follow-up). Medicare is the US’s federal health insurance program for people aged 65 and older in addition to younger people with disabilities or end-stage renal disease. Medicare covers inpatient (part A) and outpatient (part B) care as well as prescription drugs (part D) [27]. We obtained physician information from the 2013 American Medical Association Physician Professional Data Masterfile which contains demographics and training information regarding 1,001,536 US physicians.

### Patient selection

Our target population included patients with prevalent treated epilepsy who might be candidates for ASM discontinuation.

Similar to prior work, [28, 29] to identify prevalent treated epilepsy, we required at least one International Classification of Disease, Clinical Modification (ICD-CM) epilepsy/convulsion code (ICD-9 before October 1, 2015: 345.xx/780.3x; ICD-10 after October 1, 2015: G40/R56) plus at least one ASM fill in every year 2014–2016 (hence, appearance of at least three ICD codes, and sustained ASM treatment, over time; ASMs listed in Supplemental Table 1). Combining codes plus ASM fills in Medicare identified patients with epilepsy with an area under the curve 0.93, sensitivity 88%, and specificity 98% compared with reference gold standards of electronic medical records-based diagnosis by a blinded experienced neurologist using current International League Against Epilepsy guidelines [30]. ICD codes have a positive predictive value of 85–99% [31, 32]. We performed a sensitivity analysis to increase positive predictive value where we further required at least two total ICD codes for epilepsy in 2014–2016 [32, 33].

To identify beneficiaries with sustained treatment in the baseline period, we excluded those with any > 90-day gap in ASM fills 2014–2015. To approximate a population that might be eligible for discontinuation due to sustained seizure-freedom, we excluded beneficiaries with any emergency room or hospital visit listing any epilepsy/convulsion code in 2014–2015. Claims data do not definitively capture seizures, but we relied upon knowledge that two-thirds of patients with epilepsy are seizure-free on ASMs [34] plus we eliminated patients with seizure-related acute care visits and performed several sensitivity analyses described below, all to increase the fraction of well-controlled patients in our sample. To ensure data for most primary outcomes, we required that beneficiaries survived at least 90 days of 2016. To identify prevalent epilepsy, we required continuous parts A, B, and D coverage 2014–2016 up to date of death if applicable.

We included beneficiaries regardless of their reason for Medicare entitlement. Restricting to beneficiaries over age 65 would have lost the ability to assess for age effects and would have lost the important Medicare disabled population.

### Variables

Our outcome was time until the first day of a gap in ASM supply of various durations in 2016–2018 based on part D fill data. This definition used established methodology from work outside of epilepsy, which similarly used administrative claims data to evaluate the frequency of medication discontinuation [35, 36]. Furthermore, work within epilepsy has similarly used large prescription claims to capture retention rates of ASMs [37, 38] and patterns of ASM use [39]. To better represent discontinuation rather than transient nonadherence, [3] we explored a range of gap durations (30, 90, 180, and 360 days with no refill after the last day of the previous ASM fill).

We chose predictors to test hypotheses about candidate variables that may influence the chance of a discontinuation attempt and to explore a broad range of clinically relevant possible predictors. For example, patients on monotherapy ASM treatment may have lower-severity epilepsy nonadherence thus be more likely to consider discontinuation (and in the broader context of medical conditions lower disease severity or perceived need enables discontinuation decisions [40]), and a greater number of overall medications may increase the motivation for a discontinuation attempt. In contrast, epileptogenic neurological conditions might make patients and clinicians more reluctant to discontinue. For other factors, hypotheses existed in either direction. For example, specialist care may increase adherence [28] and therefore might promote continued treatment. On the other hand, specialists may be more skilled at balancing the pros and cons of long-term treatment or be more comfortable with considering discontinuation, thus increasing the chance of discontinuation. Moreover, clinicians could be appropriately reluctant to discontinue ASMs in older frail adults thus age could predict reduced discontinuation. Yet, older adults are particularly susceptible to adverse effects and drug-drug interactions, and older adults may be successfully treated at lower ASM doses, [41–44] and thus one could hypothesize that age could optimally predict increasing discontinuation attempts [45, 46].

We captured predictors such as those above, based on literature review and study team expert consensus possibly related to nonadherence [28] and discontinuation [40]. These included demographics (age, sex, race, Medicaid dual eligibility due to low income, rural ZIP code, [47] geographic region, reason for Medicare entitlement), clinical characteristics (dementia, depression, [48] Charlson comorbidity index, [49, 50] number of ASMs, total number of medications, neurologist visit in 2015, yearly office visits for epilepsy), and epileptogenic neurological conditions in 2014–2015 (stroke, traumatic brain injury, intracranial hemorrhage, central nervous system tumor, meningoencephalitis, cardiac arrest; Supplemental Table 2), focal or generalized epilepsy, [29] and refractory epilepsy, [51] in 2014–2015.

While time since epilepsy diagnosis could be another factor influencing discontinuation decisions, Medicare does not contain such a variable. To explore this concept, we extracted the date of the first ICD code for epilepsy/convulsions after January 1, 2008, after which we have complete ICD codes for this population. We then calculated years between that date, and January 1, 2016 (the date our follow-up began), to approximate duration of epilepsy. We categorized this variable into short (≤4 years), medium (4–7 years), and long (≥7 years).

We captured information from the Masterfile about the provider who prescribed the greatest number of ASM prescriptions and pill supply to each patient, based on their National Provider Index: specialty (neurology, or primary care), sex, years since medical school graduation, D.O. versus M.D., and the number of visits with that beneficiary in 2015. As the Masterfile does not contain a reliable field for “epileptologist” and does not inform board certification, we counted whether a provider saw ≥25% of their visits for a primary diagnosis of epilepsy. We then modified that definition to be either more (≥25% of visits plus ≥25 visits) or less (≥10% of visits) stringent. We captured whether providers were physician extenders using Medicare data.

### Statistical analysis

We used survival analysis to quantify time from January 1, 2016, until the first day of a gap in ASM supply of each duration. We used the Fine and Gray method [52] accounting for competing risks that could render a beneficiary ineligible for the outcome: death, emergency room or inpatient visit listing an epilepsy/convulsion code in any ICD position (suggesting the possibility of a breakthrough seizure which would render the patient ineligible for discontinuation), and losing part D coverage (preventing detection of the primary outcome). Beneficiaries were censored on January 1, 2019, minus the gap duration required for the primary outcome.

We evaluated the association between each prespecified predictor and 180-day gaps using Cox proportional hazards models with robust standard errors accounting for clustering within physicians, censored upon the above competing risks [53]. We chose 180-day gaps as the primary outcome for Cox models because adherence literature has previously considered gaps > 180 days as ‘untreated’ periods, [4] which likely represents a sufficient period to indicate discontinuation rather than transient nonadherence. We included all variables of theoretical importance, then used manual backward selection to remove variables violating the proportional hazards assumption until a global test for scaled Shoenfeld residuals no longer significantly (*p* < 0.05) interacted with time. To evaluate the model, we calculated the Harrell’s C concordance statistic [54] and performed calibration plots.

We prespecified sensitivity analyses to evaluate the robustness of our findings. 1) To further enrich our sample with well-controlled, non-refractory, adherent beneficiaries at baseline most likely to be eligible for discontinuation due to seizure-freedom after continuous treatment, we restricted to those on ASM monotherapy, with no refractory epilepsy code, and proportion of days covered over 80% in 2014–2015. The proportion of days covered represents the proportion of days with medication supply and ranges from 0% (no pill supply) to 100% (continuous pill supply). It is a widely accepted measure for claims-based definitions of adherent periods at a threshold of > 80% [28, 55]. 2) Because some drug costs for beneficiaries with dual Medicaid eligibility could be covered by Medicaid rather than Medicare thus overestimating discontinuation rates, we stratified by any dual Medicaid eligibility 2016–2018. 3) Because beneficiaries also taking an ASM for non-epilepsy diagnoses (e.g., pain, mood) may continue their ASM even if seizures are well-controlled for their non-epilepsy indication, we restricted to the most common ‘pure’ ASMs (levetiracetam, phenytoin, phenobarbital) that would be less likely to pose this issue. 4) We explored the influence of ‘epileptologist’ across different definition thresholds as described above. 5) To be maximally sensitive at excluding patients after breakthrough seizures, our primary censorship procedure involved censoring patients upon the first acute care visit with any ICD code for seizures or epilepsy. However, because acute visits could list epilepsy in an ICD code position as a relevant comorbidity despite not actually having had a seizure on that day (i.e., sensitive but not specific), to increase specificity we modified our censorship procedure to consider only emergency room or inpatient visit listing an epilepsy/convulsion code in the primary ICD position.

We also performed one secondary analysis to evaluate the correlation between time since diagnosis and 180-day gaps. Because absence of a diagnostic code could represent either absence of a diagnosis versus a beneficiary who had not yet enrolled in Medicare, we restricted to beneficiaries continuously enrolled in parts A and B between 2008 and 2016.

Given predictors could be correlated, we calculated variance inflation factors for each predictor, which represent the relative amount by which collinearity with other predictors increases a predictor’s variance. A common “rule of thumb” is that a value less than 10 is acceptable [56].

Data were analyzed using SAS 9.4 (Cary, NC) and Stata 16.0 (College Station, TX).

## Results

**Fig. 1:**
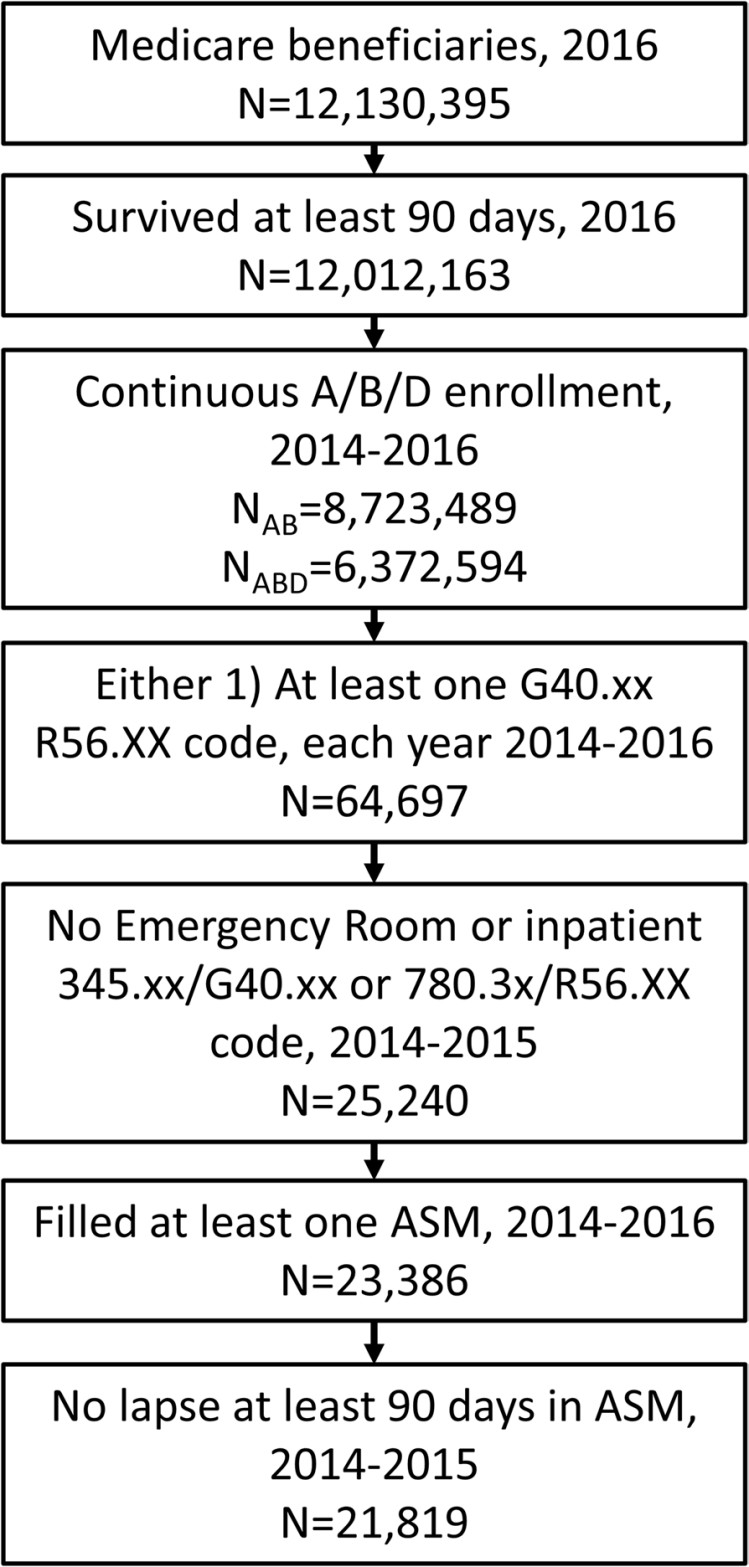
Patient flowchart

Among 21,819 beneficiaries (Fig. 1), the median age was 58 (min 20, IQR 46–71, max 101; 39% were at least 65 years old), 50% were female, 84% were white, and 62% were dual eligible for Medicaid (Table 1).

**Table 1:** Population description (*N* = 21,819)

		Median or N (interquartile range or %)
**Age**		58 (46–71)
**Female sex**		10,914 (50%)
**Race**^**a**^	White	17,809 (84%)
	Black	2510 (12%)
	Hispanic	670 (3%)
	Asian	257 (1%)
**Dual eligible for Medicaid**		13,717 (63%)
**Rural ZIP code**		6231 (29%)
**Reason for entitlement**	Disability	13,229 (61%)
	Age	8581 (39%)
	End-stage renal disease	42 (< 1%)
**Region**	South	8230 (39%)
	Midwest	5644 (27%)
	Northeast	4075 (19%)
	West	3225 (15%)
**Any neurologist visit, 2015**		15,133 (69%)
**Any primarily epilepsy visit, 2015**		17,203 (79%)
**Unique medications (No.), 2015**		8 (5–12)
**Unique ASMs (No.), 2015**		1 (1–2)
**ASM proportion of days covered (%), 2014–2015**		92% (81–96%)
**Older generation ASM, 2015**		12,517 (57%)
**Brand name ASM, 2015**		5430 (25%)
**Total part D out of pocket cost, 2015**		$66 ($0–$301)
**Epilepsy type**^**b**^	Focal	6046 (28%)
	Generalized	4270 (20%)
	Both	2169 (10%)
	Neither	9334 (43%)
**Refractory epilepsy, 2014–2015**		4445 (20%)
**Epileptogenic neurological conditions, 2014–2015**	Ischemic stroke	2160 (10%)
	Traumatic brain injury	563 (3%)
	Intracranial hemorrhage	516 (2%)
	Tumor	454 (2%)
	Meningoencephalitis	109 (< 1%)
	Cardiac arrest	49 (< 1%)
**Dementia**		1762 (8%)
**Depression**		6250 (29%)
**Charlson comorbidity index, 2015**	0	12,938 (59%)
	1–3	7968 (37%)
	4–6	788 (4%)
	7+	125 (1%)
**Acute care visits, 2015**^**c**^	0	17,005 (78%)
	1	3267 (15%)
	2+	1547 (7%)
**Primary ASM prescriber, 2015**^**d**^	Neurologist	11,242 (59%)
	Epileptologist	430 (2%)
	Primary care physician	6872 (36%)
	Female	4315 (23%)
	Years since med school	27 (19–34)
	Physician extender	1832 (9%)
	D.O.	1515 (8%)
	# visits this patient, 2015	2 (1–3)

*ASM* Antiseizure medication

^a^Race: This is how Medicare classifies race, with Hispanic as a separate category without distinguishing non-Hispanic White versus Hispanic White versus non-Hispanic Black

^b^Epilepsy type: at least one *International Classification of Diseases* code for focal and/or generalized epilepsy

^c^Acute care visits: we excluded beneficiaries with epilepsy-related acute care visits, thus this variable refers to any non-epilepsy acute care visit

^d^Primary ASM prescriber: the single physician who prescribed the greatest number of antiseizure medication prescriptions and pill days in 2015. For the main definition, ‘epileptologist’ was defined as at least 25% of a provider’s Evaluation/Management codes being primarily for epilepsy, though we performed several sensitivity definitions of this (less restrictive: at least 10%; more restrictive: at least 25% plus at least 25 visits in the year). Physician extender was defined as Nurse Practitioner or Physician Assistant

The following competing risks occurred before the occurrence of a 180-day gap: 9957 (46%) had at least one emergency room or inpatient visit listing epilepsy or seizures in any ICD position (median 362 days to the first visit, IQR 164–661), 358 (2%) died (median 531 days until death, IQR 346–758), and 179 (1%) had at least 1 month without continuous part D coverage (median 700 days to first loss of coverage, IQR 487–821). There were 2536 (12%) beneficiaries with at least one emergency room or inpatient visit listing epilepsy or seizures in the primary ICD code position (median 464 days, IQR 209–752).

Overall, 5191 (24%) had a 30-day ASM gap, 1753 (8%) had a 90-day gap, 834 (4%) had a 180-day gap, and 381 (2%) had a 360-day gap.

**Fig. 2:**
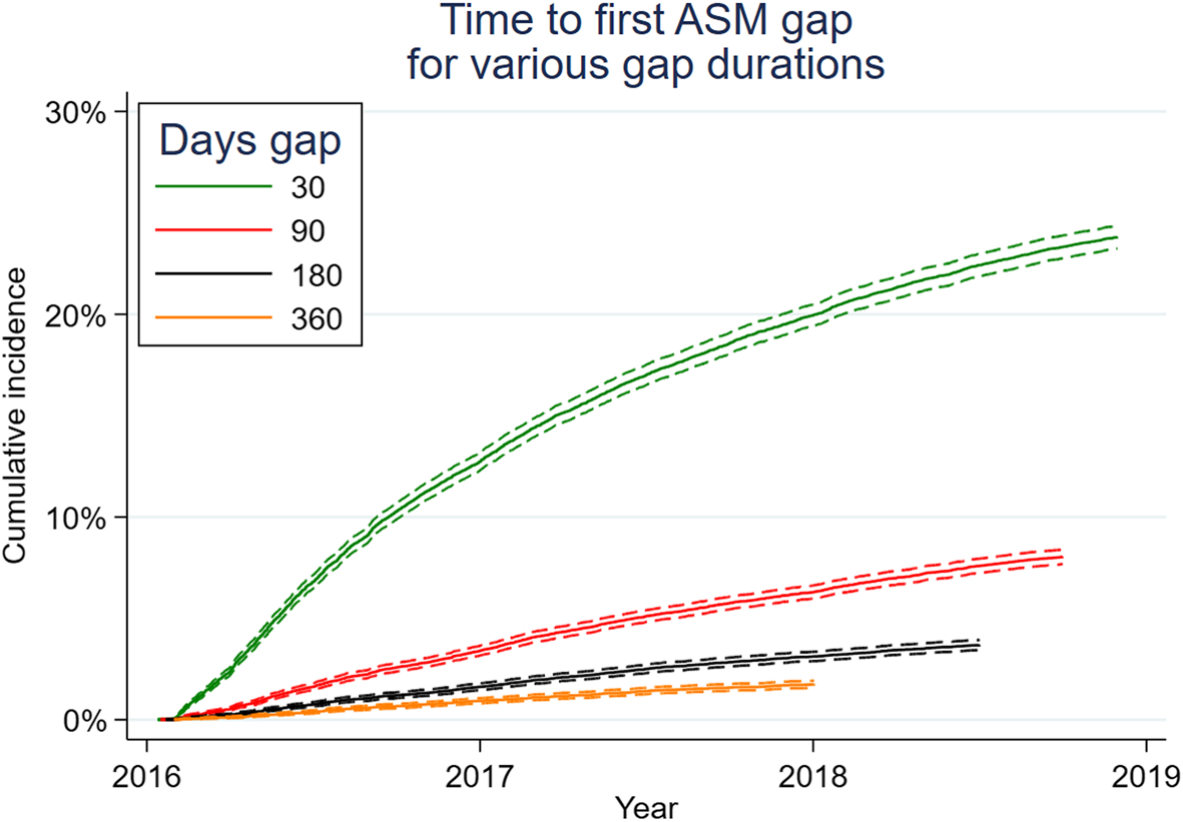
Cumulative incidence functions of antiseizure medication gaps of increasing durations. Legend: The outcome was time to the first day of the first gap in antiseizure medication pill supply of each specified number of days. The outcome was censored if a competing risk occurred before a fill gap - death, emergency room or inpatient visit listing seizures as a diagnosis, or losing part D coverage. Overall, 5191 (24%) had a 30-day ASM gap, 1753 (8%) had a 90-day gap, 803 (4%) had a 180-day gap, and 381 (2%) had a 360-day gap. Curves do not extend until 1/1/2019 because a sufficient number of days were required to evaluate the outcome (e.g. 30, 90, 180, or 360 days before 1/1/2019). On 1/1/2018, at which point all outcomes were measurable, cumulative incidences were 20, 6, 3, and 2% for 30-, 90-, 180-, and 360-day gaps, respectively. Dashed lines represent 95% confidence intervals

Figure 2 displays cumulative incidence functions. On January 1, 2018, at which point all outcomes were measurable, cumulative incidences were 20, 6, 3, and 2% for 30-, 90-, 180-, and 360-day gaps, respectively.

Among those 5191 with a 30-day gap, 4800 (92%) had a subsequent ASM fill during the 2016–2018 observation window. Percentages for other gap durations were 1301/1753 (74%) for 90-day gaps, 405/803 (50%) for 180-day gaps, and 102/381 (27%) for 360-day gaps.

In our sensitivity population definition, among the 19,010 (87%) with at least two ‘epilepsy’ (345.xx/G40.xx) ICD codes in addition to ASM fills during each of the baseline years (2014–2016), outcomes were nearly identical to the primary analysis: 24, 8, 4, and 2%, respectively.

**Fig. 3:**
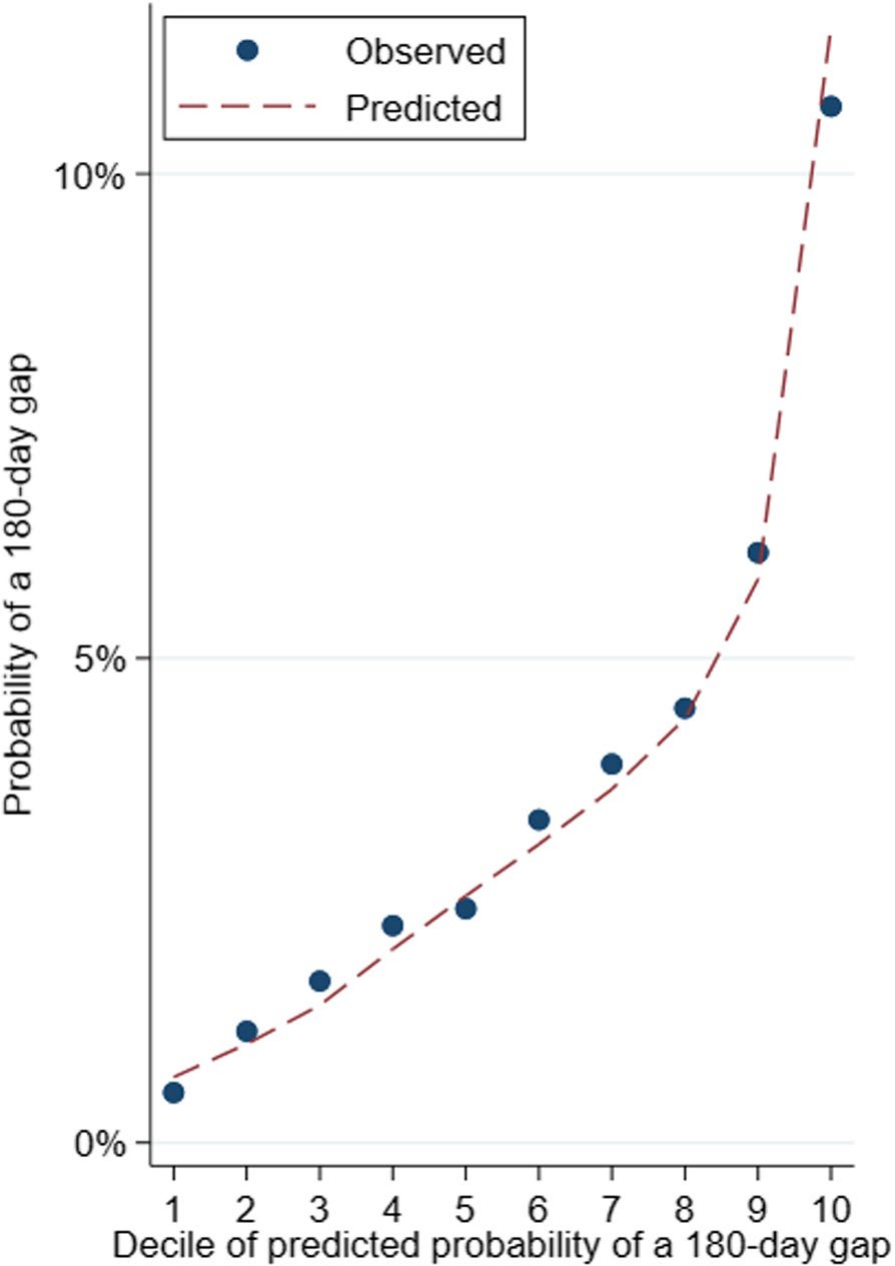
Cox proportional hazards model calibration plot. Legend: Observed and predicted probabilities for 180-day gaps were similar

Our Cox regression demonstrated moderate concordance (0.72), good calibration (Fig. 3), and acceptably low variance inflation factors for all predictors (Supplemental Table 3). Table 2 displays adjusted predictors of 180-day gaps. Numerous variables predicted increased chance of a 180-day gap: number of unique medications in 2015 (hazard ratio [HR] 1.03 per medication, 95% confidence interval [CI] 1.01–1.05), depression (HR 1.26, 95% CI 1.06–1.50), meningoencephalitis (HR 2.37, 95% CI 1.08–5.24), and epileptologist prescribing physician (HR 2.37, 95% CI 1.39–4.03). Other variables predicted decreased 180-day gaps: Medicaid dual eligibility (HR 0.75, 95% CI 0.60–0.95), number of unique ASMs in 2015 (e.g., 2 versus 1: HR 0.37, 95% CI 0.30–0.45), greater baseline adherence (> 80% versus ≤80% of days in 2015 with ASM pill supply: HR 0.38, 95% CI 0.32–0.44), and older generation ASM (HR 0.82, 95% CI 0.69–0.98). Age was not a significant adjusted predictor (per decade: HR 0.97, 95% CI 0.88–1.07).

**Table 2:** Associations between each variable and a 180-day gap, *N* = 17,385

		Hazard ratio*	95% confidence interval
**Age, per decade**		0.97	0.88–1.07
**Female sex**		1.08	0.91–1.26
**Race**	White	Reference	Reference
	Black	1.20	0.94–1.53
	Hispanic	1.23	0.78–1.95
	Asian	**1.74**	**1.02–2.98**
**Dual eligible for Medicaid**		**0.75**	**0.60–0.95**
**Rural ZIP code**		0.98	0.82–1.18
**Reason for entitlement**	Age^a^	1.17	0.88–1.54
**Region**	South	Reference	Reference
	Midwest	0.85	0.69–1.05
	Northeast	0.85	0.67–1.08
	West	1.02	0.80–1.29
**Any neurologist visit, 2015**		0.90	0.72–1.12
**Any primarily epilepsy visit, 2015**		0.88	0.73–1.07
**Unique medications, 2015**		**1.03**	**1.01–1.05**
**Unique ASMs (No.), 2015**	1	Reference	Reference
	2	**0.37**	**0.30–0.45**
	3+	**0.23**	**0.16–0.33**
**ASM proportion of days covered > 80%, 2014–2015**		**0.38**	**0.32–0.44**
**Older generation ASM, 2015**		**0.82**	**0.69–0.98**
**Total Part D out of pocket cost (per $100), 2015**		1.00	0.99–1.01
**Epilepsy type**	Focal	Reference	Reference
	Generalized	1.00	0.78–1.27
	Both	0.76	0.54–1.06
	Neither	1.00	0.82–1.23
**Refractory epilepsy**		0.96	0.76–1.21
**Epileptogenic neurological conditions, 2014–2015**	Ischemic stroke	1.26	0.98–1.61
	Traumatic brain injury	0.76	0.44–1.31
	Intracranial hemorrhage	1.21	0.79–1.86
	Tumor	1.30	0.79–2.13
	Meningoencephalitis	**2.37**	**1.08–5.24**
	Cardiac arrest	0.56	0.16–2.00
**Dementia**		0.96	0.71–1.29
**Depression**		**1.26**	**1.06–1.50**
**Charlson comorbidity index, 2015**	0	Reference	Reference
	1–3	0.92	0.76–1.10
	4–6	1.20	0.82–1.76
	7+	1.36	0.68–2.69
**Acute care visits for any condition, 2015**	0	Reference	Reference
	1	1.06	0.85–1.33
	2+	1.06	0.79–1.42
**Primary ASM prescriber**	Neurologist	1.10	0.89–1.35
	Epileptologist	**2.37**	**1.39–4.03**
	Female	1.05	0.86–1.27
	Decades since med school	0.99	0.91–1.08
	Physician extender^b^	1.01	0.77–1.34
	D.O.	1.11	0.74–1.33
	# visits this patient, 2015	**1.03**	**1.00–1.06**

*ASM* Antiseizure medication

*Hazard ratios are adjusted for all other variables contained in this table. Variables that appear in Table 1 but not Table 2 were omitted due to non-proportional hazards, unless mentioned below. Bolded hazard ratios are significant at *p* < 0.05

^a^End-stage renal disease was omitted due to unstable estimates with a small sample size and thus essentially reason for entitlement of age is essentially being compared with reason for entitlement of disability

^b^Hazard ratios for primary ASM prescriber were all computed in this model including only beneficiaries whose primary ASM prescriber was a physician, given data from the Physician Masterfile as covariates. Thus, the main model did not include ‘physician extender’ as a covariate, but we reran the model omitting physician variables and including ‘physician extender’ as a variable (*N* = 19,729) with little meaningful change to other reported coefficients

**Fig. 4:**
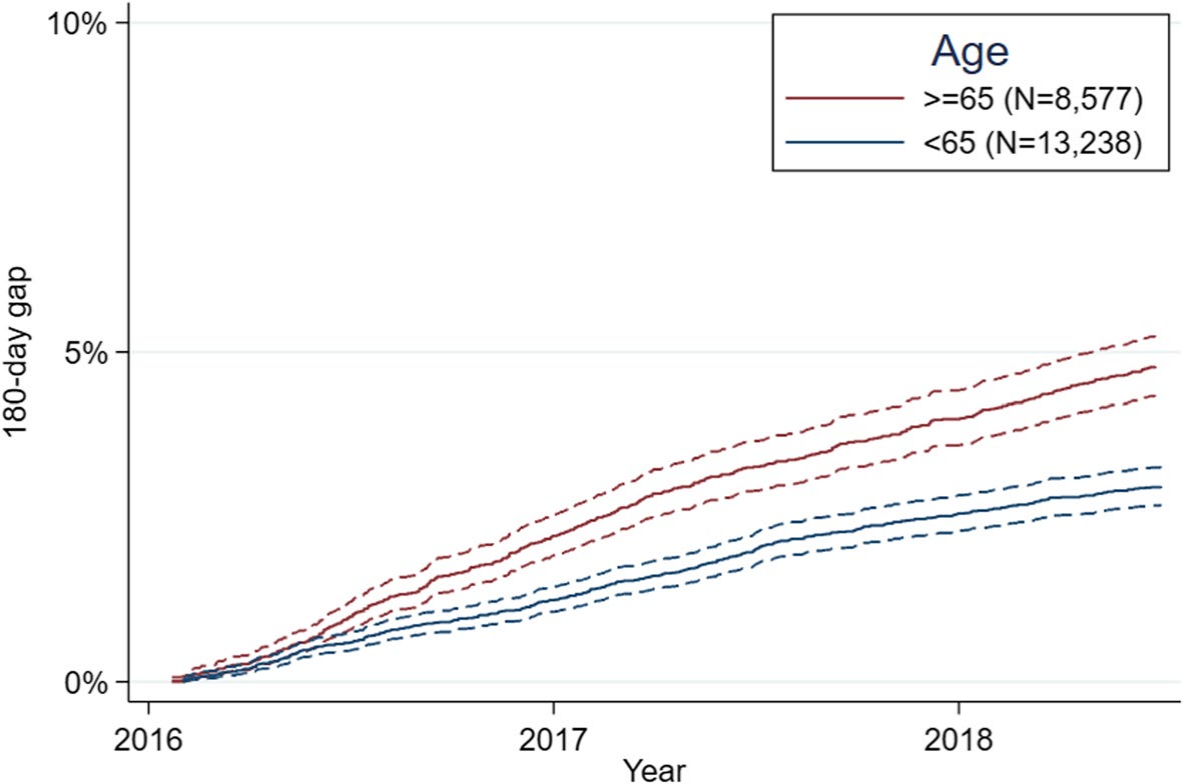
Cumulative incidence functions stratified by age

Absolute effects were all small. For example, the frequency of 180-day gaps for selected comparisons were: 1) 5% versus 3% for age at least 65 years versus less than 65 (Fig. 4), 2) 5, 2, and 1% for beneficiaries on one, two, or at least three ASMs, 3) 3% versus 6% for those with PDC at least 80% versus less than 80%, and 4) 4% regardless of whether a beneficiary’s main prescriber was an epileptologist (though this was significant in adjusted analysis, Table 2).

We performed additional sensitivity analyses:

- First, among the 7672 (35%) with no refractory epilepsy code and proportion of days covered over 80% on monotherapy at baseline, the frequency of gaps of each duration was similar to the primary analysis (26, 8, 4, and 2%, for 30-, 90-, 180-, and 360-day gaps, respectively; cumulative incidence functions essentially identical, not displayed). Among those with a refractory code 2.47% had a 180-day gap, and among those without a refractory code 3.99% had a 180-gap (unadjusted *p* < 0.01), though per Table 2 refractory epilepsy was not a significant adjusted predictor.
- Second, having any dual Medicaid eligibility in 2016–2018 was associated with lower cumulative incidence of each gap (*p* all < 0.01). The probability of 30-, 90, 180-, and 360-day gaps for beneficiaries with versus without dual eligibility were: 19% versus 33, 6% versus 11, 3% versus 5, and 1% versus 2%, respectively.
- Third, we restricted to the top three ‘pure’ ASMs. Probabilities of 180-day gaps were 6% for levetiracetam, 5% for phenytoin, and 3% for phenobarbital.
- Fourth, when modifying the definition of ‘epileptologist’ to be less stringent (at least 10% of visits primarily for epilepsy), ‘epileptologist’ in a regression otherwise identical to Table 2 was no longer significant (HR 0.88, 95% CI 0.61–1.29). When modifying the definition to be more stringent (at least 25% of visits primarily for epilepsy plus at least 25 visits primarily for epilepsy), ‘epileptologist’ remained significant (HR 2.25, 95% CI 1.26–4.02).
- Fifth, when modifying our censorship procedure to consider the first acute visit listing epilepsy/seizures in the primary ICD code position (as opposed to any position), 7036 (32%) had a 30-day ASM gap, 2427 (11%) had a 90-day gap, 1118 (5%) had a 180-day gap, and 531 (2%) had a 360-day gap before censorship.

**Fig. 5:**
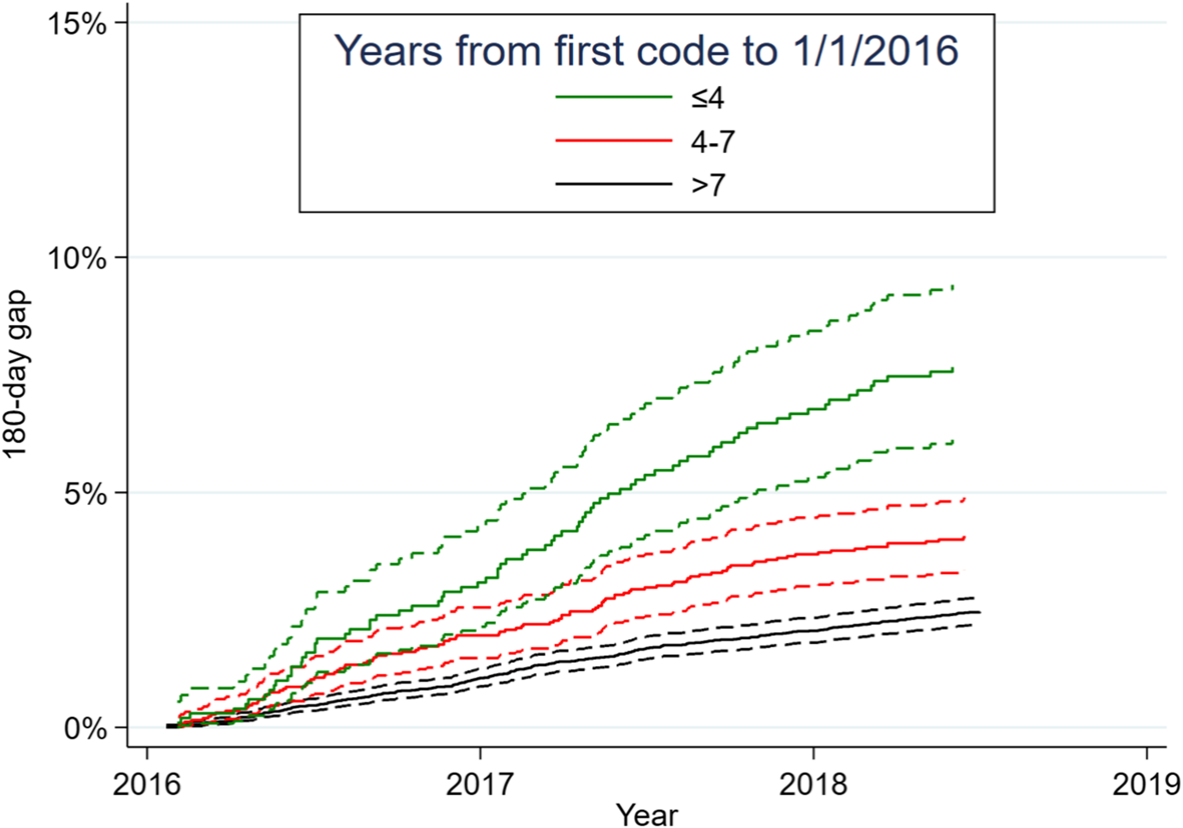
Cumulative incidence functions stratified by time since first epilepsy diagnosis. Legend: Time since first epilepsy diagnosis was defined as the number of years between the first epilepsy/convulsion code after January 1, 2008 (the greatest lookback period available in our data) until January 1, 2016 (the start of our cohort’s follow-up). This analysis was restricted to the 14,697 beneficiaries with continuous parts A and B Medicare enrollment in 2008–2016

We performed a secondary exploratory analysis correlating time since diagnosis, and whether beneficiaries had a 180-day gap, among 14,697 beneficiaries with continuous Medicare starting January 1, 2008. Among beneficiaries whose first epilepsy/convulsion ICD code appeared within ≤4 years, 4–7 years, and ≥ 7 years of January 1, 2016, respectively, the prevalence of 180-gaps were: 77/1005 (8%), 104/2552 (4%), and 274/11,140 (2%) (*p* < 0.01). Figure 5 demonstrates that cumulative incidence of 180-day gaps was highest for the group with the shortest time since diagnosis.

## Discussion

In this Medicare population with prevalent treated epilepsy without recent epilepsy-related acute care utilization, sustained ASM gaps were extremely rare. These findings were robust to numerous sensitivity analyses where we modified the population and analytic method. Our main finding contrasts with guidelines suggesting that seizure-free patients may consider ASM discontinuation after 2 years of seizure-freedom [16] combined with the expectation that two-thirds of patients will be seizure-free on ASMs [34] and literature supporting that approximately 70% of patients will remain seizure-free even post-discontinuation [18]. While the optimal discontinuation rate is not known, our results raise the possibility that this older population with heightened vulnerability to medication-related harms may be overtreated.

While all absolute effects were small, these data supported some but not all our hypotheses regarding predictors of gaps. For example, a lower number of ASMs and an increased overall medication number did predict an increased chance for gaps. This is not surprising, as lower-severity epilepsy and increasing overall medical treatment burden could both encourage patients and physicians to be increasingly judicious about the necessity of ASMs. Additionally, depression increased the chance of gaps, which may fit with prior literature suggesting decreased medication adherence among patients with depression [57]. However, other hypotheses were not supported. For example, we hypothesized that epileptogenic neurologic conditions might discourage gaps by virtue of increasing future relapse risk due to a neurological lesion. However, comorbidities such as hemorrhage, stroke, or traumatic brain injury were nonsignificant. One possibility could be that nonsignificant results for those variables reflect the fact that in our dataset patients were all either disabled or older, thus perhaps replicating our study in other private insurance datasets might find differences where we did not. One may also have hypothesized that patients on older generation ASMs should be most likely to consider an ASM discontinuation attempt given older generation ASMs are most likely to have drug-drug interactions systemic adverse effects [46]. Yet, we found that older generation ASMs predicted decreased gaps which could further suggest underutilization of discontinuation attempts. Also, beneficiaries with the most recent diagnosis were more likely to have a 180-day gap. Shorter duration of epilepsy could confer lower risk of relapse [58] leading to increased discontinuation. Alternatively, longer duration of treatment could increase inertia against considering discontinuation (“status quo bias”), [59, 60] thus future work disentangling the degree to which biological risk versus cognitive biases influence decision-making would be quite interesting, and future work using electronic medical record-based with higher quality measurement of duration of epilepsy could seek to confirm our results.

Regarding another hypothesis, an epileptologist prescribing physician doubled the chance of discontinuation. This could suggest increased comfort with discontinuation decisions among subspecialists, even conditioned upon the chance that epileptologists treat patients with more severe disease. Interestingly, whereas ‘epileptologist’ was associated with ASM gaps, ‘neurologist’ was not, and we also did not find a ‘duration of practice’ effect. Other work did find that fewer years in practice predicted a lower chance of recommending ASM withdrawal in response to hypothetical vignettes, [61] though one advantage of our study is that we examined real-world outcomes rather than hypothetical recommendations.

It may be quite reasonable in many circumstances to be reluctant about withdrawing ASMs. In a patient who is tolerating their ASM without difficulty, and particularly if they have had a previous unsuccessful discontinuation attempt, long-term treatment is sensible. Our population particularly contained many older adults, for whom clinicians may be reluctant to withdraw ASMs in the context of cerebrovascular disease or structural brain abnormalities. There are other challenges when considering discontinuation. A freely available rapid point-of-care post-withdrawal seizure risk calculator exists which demonstrated moderate discrimination during development [58, 62]. However, a more recent study found the calculator demonstrated poor external accuracy, [63] and the existing calculator shows only post-withdrawal risk leaving individualized absolute treatment effects sometimes unclear. Critically, no work to date clearly informs any particular risk threshold above or below which withdrawal is known to result in benefit versus harm, and thus existing literature could be insufficient to guide candidates for withdrawal explaining reluctance as has been pointed out by prior Delphi methods surrounding deprescribing in older adults [64]. Furthermore, medicolegal implications of a patient having a recurrent seizure may vary across contexts. For example, driving privileges vary across states and countries, which could discourage ASM withdrawal according to each context.

Ultimately, our study motivates the importance of future implementation science and risk prediction methods to inform whether our data truly reflect widespread overtreatment, versus whether clinicians and patients are appropriately more reluctant than guidelines have suggested discontinuing ASMs. Importantly, the most recent guidelines on ASM discontinuation no longer recommends any single time-based threshold after which to consider discontinuation, but rather expresses considerable uncertainty regarding risk factors and outcomes underscoring the importance of further research [17].

Our study has numerous limitations.

First, fill gaps could overestimate discontinuation attempts. A beneficiary might falsely appear to have a ‘gap’ if they began cutting pills in half due to a dose reduction making their bottle last longer than originally planned. Though, if we overestimated the true rate of discontinuation that would only reinforce our conclusions.

Second, prescription fill gaps could underestimate discontinuation attempts if a patient decided to stop their ASMs and then had a seizure during follow-up before our time-based gap definitions. However, on average, seizure relapse rates are only approximately 30% [18] by two to 5 years post-discontinuation. Thus, this seems unlikely to be the only explanation for such low sustained discontinuation rates. Including patients with ongoing seizures could have also underestimated discontinuation attempts by increasing the denominator. The absolute effect of this bias is likely small though. Even if we had actually ended up including all epilepsy patients on an ASM, only two-thirds of whom may be seizure-free [34], the true denominator would have been two-thirds of what we included, hence the true proportion of 180-day gaps would be maximally 4% * (3/2) = 6%, instead of 4% as we found.

Third, claims do not distinguish reasons for gaps, whether patient nonadherence versus physician-directed “deprescribing.” Regardless, our work still provides prognostic insight into the probability that patients treated with ASMs thereafter achieve sustained ASM-freedom. Also, our finding that 24% of beneficiaries had at least a 30-day gap was consistent with other literature reporting nonadherence rates ~ 20–50% [65, 66]. Nonadherence is associated with increased acute care utilization [4]. Future work may focus on whether initial nonadherence is associated with an increased chance of future discontinuation, in addition to the association specifically between ASM withdrawal and healthcare utilization in well-controlled epilepsy.

Fourth, ICD codes could misclassify epilepsy. False positives could occur if clinicians attach epilepsy or convulsion codes to patients with psychogenic seizures or acute symptomatic seizures. False negatives could occur if patients with epilepsy do not seek care or do but are underdiagnosed. Nevertheless, evidence [32] supports 85–100% positive predictive value (i.e. false positive rate of 0–15%) when using ICD codes, positive predictive value is further improved by requiring ASM treatment as we did in our main analysis, [32] and our population sensitivity analyses did not change conclusions. Furthermore, claims could misclassify epilepsy type. Compared with electronic health records, ICD codes for focal versus generalized/unknown epilepsy achieved a sensitivity of 70% and specificity of 79% [29]. Future work using electronic health records enabling chart-based verification of epilepsy diagnosis and type may complement our study.

Fifth, while we captured many relevant variables (including age, focality, epileptogenic neurological conditions, dementia, comorbidity burden, neurologist care, ASM treatment at least 2 years, etc.), Medicare lacks potentially important variables such as total duration of ASM treatment, prior failed discontinuation attempts, EEG results, or employment and driving considerations [22, 23] (possibly mitigated here by considering a disabled/older population). We also acknowledge that complex interactions may exist between variables that were not studied here. Though, we sought to include terms in our model only with a priori theoretical justification, rather than further exploratory analysis of hypothesis-generating interactions.

Sixth, this older/disabled Medicare population may not generalize to other populations with less polypharmacy, poorer insurance, or different socioeconomic or medical consequences of seizures. Sixty-one percent of our population qualified for Medicare due to disability. Nationally representative work demonstrates that people with epilepsy have three times as many physical, mental, and social limitations compared with the general population [67]. Seizures may prevent a patient from working or driving, but also patients with epilepsy have increased prevalence of most other chronic pain, psychiatric, and general medical conditions which all may explain high rates of disability [68]. In an ad-hoc analysis, there were differences in our sample between the group qualifying for Medicare due to disability versus the remainder of our sample qualifying predominantly for Medicare due to older age. For example, the group qualifying due to disability had a lower mean Charlson index (0.7 (standard deviation (SD) 1.4)) versus 1.0 (SD 1.2)) and were more likely to be dual Medicaid eligibility (86% versus 28%) or to have an epilepsy-related visit in 2015 (83% versus 72%), all *p* < 0.05 via Chi-squared or t-tests. These differences emphasize the importance of adjusting for such variables in our models as we have done, and future work may seek to reproduce our findings in other data sources.

Despite these limitations, Medicare data has many strengths. Medicare covers ~ 20% of the US population [69] and as above part D provides prescription coverage for 48 million lives. This data source applies a far greater reach to the US population than would be possible with any single institutional dataset, registry, or most any private insurance database. Medicare also enables linkage with detailed physician information via the Masterfile, provides complete capture of pharmacy fills including the exact fill dates and quantities with high-quality longitudinal follow-up, and particularly enables studying older adults where polypharmacy is most applicable. That said, our work should be complemented in the future by prospective studies with increased granularity.

## Conclusions

In this Medicare population, sustained periods of ASM gaps occurred less frequently than seems endorsed by guidelines. Seeing an epileptologist may increase the chance of considering discontinuation whereas other patient factors decreased the chance of discontinuation (e.g., greater number of ASMs, greater baseline adherence), but absolute differences were small. Future work is needed to explore barriers and facilitators driving why ASM discontinuation appears to be occurring less frequently than suggested by past guidelines and to better enable physicians to weigh the risks and benefits of treatment.

## Supplementary Information

**Additional file 1: Supplemental Table 1.** List of antiseizure medications considered to define the cohort, sorted by percent of all pill days in this population in 2016. **Supplemental Table 2.** International Classification of Diseases (ICD) codes. **Supplemental Table 3.** Evaluation of collinearity for variables in our Cox model.

## Data Availability

Medicare data are not publicly available due to data use agreement restrictions but are available for purchase (https://resdac.org/).

## Abbreviations

ASM: Antiseizure medication
CI: Confidence interval
IQR: Interquartile range
ICD: International Classification of Diseases
