# Supplementary material for: Incidence of and predictors for antiseizure medication gaps in Medicare beneficiaries with epilepsy: a retrospective cohort study
Source: BMC Neurol. 2022 Sep 1;22:328. doi: 10.1186/s12883-022-02852-6 (PMC9434838; doi:10.1186/s12883-022-02852-6)
Supplement: Supplementary file 1 — Additional file 1: Supplemental Table 1. List of antiseizure medications considered to define the cohort, sorted by percent of all pill days in this population in 2016. Supplemental Table 2. International Classification of Diseases (ICD) codes. Supplemental Table 3. Evaluation of collinearity for variables in our Cox model. [file 12883_2022_2852_MOESM1_ESM.pdf]

Supplemental Table 1: List of antiseizure medications considered to define the cohort, sorted by percent of all pill days in this population in 2016.

Antiseizure medication	Percent of all pill days in 2016
Levetiracetam	22%
Phenytoin	13%
Lamotrigine	11%
Valproate	11%
Carbamazepine	11%
Topiramate	6%
Gabapentin	6%
Phenobarbital	5%
Lacosamide	3%
Oxcarbazepine	3%
Zonisamide	2%
Primidone	2%
Pregabalin	1%
Clobazam	1%
Felbamate	0.3%
Eslicarbazepine	0.3%
Rufinamide	0.2%
Ethosuximide	0.2%
Tiagabine	0.2%
Perampanel	0.1%
Ezogabine	<0.1%
Methsuximide	<0.1%
Brivaracetam	<0.1%
Vigabatrin	<0.1%
Everolimus	<0.1%
Ethotoin	<0.1%
Adrenocorticotropic hormone	0%
Cannabidiol	0%
Cenobamate	0%
Fenfluramine	0%
Stiripentol	0%

Supplemental Table 2: International Classification of Diseases (ICD) codes

	**ICD-9 (before 10/1/2015)**	**ICD-10 (after 10/1/2015)**
Cardiac arrest	427.41, 427.42, 427.5X	I46.XX, Z86.74
Central nervous system tumor	191.XX, 239.6X, 198.3X	C71.XX
Dementia	290.0X, 290.1X, 290.2X, 290.3X, 290.4X, 294.0X, 294.1X, 294.2X, 294.8X, 331.0X, 331.2X, 331.7X, 331.82, 797.XX	F03.90, F01.50, F01.51, F04.XX, F02.80, F02.81, F03.91, F06.1X, F06.8X, G30.0X, G30.1X, G30.8X, G30.9X, G31.0X, G31.1X, G94.XX, G31.83, R41.81
Depression	296.20-296.25, 296.30-296.35, 296.5X, 296.6X, 296.82, 296.90, 300.4X, 309.0X, 309.1X, 309.28, 311.XX	F313.XX-F316.XX, F32.0X-F32.9X, F33.0X-F33.9X, F34.1X, F34.8X, F34.9, F38.0X, F38.1X, F38.8, F39.XX, F41.2X, F99.XX
Epilepsy/convulsions	-Epilepsy: 345.xx<br>-Convulsions: 780.3x	-Epilepsy: G40.XX<br>-Convulsions: R56.XX
Focal/generalized epilepsy	-Focal: 345.4X, 345.5X, 345.7X<br>-Generalized: 345.0X, 345.1X, 345.6X	-Focal: G40.0X, G40.1X, G40.2X<br>-Generalized: G403.x, G404.x, G40.AX, G40.BX, G40.81X, G40.82X
Intracranial hemorrhage	430.XX, 431.XX, 432.XX	I60.XX, I61.XX, I62.XX, I69.0X, I69.1X, I69.2X, S06.4X, S06.5X, S06.6X
Ischemic stroke	433.01, 433.11, 43321, 433.31, 433.81, 433.91, 434.01, 434.11, 434.91, 436.XX	I63.XX, I69.3X
Meningoencephalitis	320.XX, 321.XX, 322.XX, 323.XX	G04.0X, G04.2X, G04.3X, G04.81, G04.90, G03.XX
Refractory epilepsy	345.01, 345.11, 345.41, 345.51, 345.61, 345.71, 345.81, 345.91	G40.01, G40.11, G40.21, G40.31, G40.A1, G40.B1, G40.41, G40.803, G40.804, G40.813, G40.814, G40.823, G40.824, G40.91
Traumatic brain injury	800.XX, 801.XX, 802.XX, 803.XX, 804.XX, 850.XX	S02.0X, S02.1X, S02.8X, S02.91, S04.02, S04.03, S04.04, S06.XX, S07.1

Supplemental Table 3: Evaluation of collinearity for variables in our Cox model.

Variable	Variance inflation factor*
Age, decades	3.43
Reason for entitlement: old age	3.38
Dual eligible for Medicaid	2.15
Any neurologist visit, 2015	1.81
Neurologist prescriber	1.70
Unique medications (No.), 2015	1.62
Total part D out of pocket cost, 2015	1.46
Charlson comorbidity index, 2015	1.34
Any epileptologist visit, 2015	1.28
Focal epilepsy type	1.27
Unique ASMs at least 2, 2015	1.24
Refractory epilepsy, 2014-2015	1.22
Older generation ASM	1.19
Prescriber, # visits this patient, 2015	1.16
Ischemic stroke, 2014-2015	1.15
Depression, 2014-2015	1.15
Acute care visits, 2015	1.13
Prescriber, decades since medical school	1.13
Dementia, 2014-2015	1.10
White race	1.08
Prescriber, female	1.08
Female sex	1.06
South region	1.05
Intracranial hemorrhage, 2014-2015	1.05
Rural ZIP code	1.04
Mean possession ratio at least 80%	1.04
Prescriber, D.O.	1.04
Prescriber, epileptologist	1.03
Traumatic brain injury	1.02
CNS tumor, 2014-2015	1.01
Meningoencephalitis, 2014-2015	1.00
Cardiac arrest, 2014-2015	1.00

*Variance inflation factors represent the relative amount by which collinearity increases a predictor’s variance. A common “rule of thumb” is that a value less than 10 is acceptable.
